# Pulmonary congestion and systemic congestion in hemodialysis: dynamics and correlations

**DOI:** 10.3389/fneph.2024.1336863

**Published:** 2024-02-23

**Authors:** Saleh Kaysi, Bakhtar Pacha, Maria Mesquita, Frédéric Collart, Joëlle Nortier

**Affiliations:** ^1^ Nephrology Department, Brugmann University Hospital, Université libre de Bruxelles, Brussels, Belgium; ^2^ Laboratory of Experimental Nephrology, Faculty of Medicine, Université libre de Bruxelles, Brussels, Belgium

**Keywords:** hemodialysis, pulmonary congestion, lung ultrasound, dry weight, systemic congestion

## Abstract

**Introduction:**

Systemic congestion and pulmonary congestion (PC) are common in hemodialysis (HD) patients. However, the relationship between these two entities is not quite clear. We study this relationship and attempt to uncover the factors that may affect it considering different inter-dialytic intervals.

**Methods:**

A prospective pilot observational and interventional study including 18 HD patients was conducted. The following were obtained: i) B-line score (BLS) by lung ultrasound (LUS) (reflecting significant pulmonary congestion if BLS > 5), ii) echocardiography, iii) bioelectrical impedance analysis (BIA) (reflecting global volume status), and iv) inferior vena cava (IVC) dynamics (reflecting systemic congestion) before and after the first two consecutive HD sessions of the week, with different inter-dialytic intervals (68 hours and 44 hours). Serum N-terminal pro-brain natriuretic peptide type B (NT-proBNP) levels were obtained before each session. Then, patients were randomized into two groups: the active group, where dry weight was reduced according to BLS + standard of care, and the control group, where dry weight was modified according to standard of care. All the measures were repeated on day 30.

**Results:**

We found no correlation between pulmonary congestion represented by BLS and IVC dimensions and dynamics reflecting systemic congestion, independent of different inter-dialytic intervals. Pulmonary congestion was quite prevalent, as mean pre- and post-dialysis BLSs were quite elevated (16 ± 5.53 and 15.3 ± 6.63, respectively) in the first session compared with the second session (16.3 ± 5.26 and 13.6 ± 5.83, respectively). Systolic (left ventricular ejection fraction) and diastolic cardiac function (e/è ratio) parameters from one side and pulmonary congestion (BLS) from the other were not always correlated. BLS was correlated to e/è ratio before HD (session 1) (*R* ^2^ = 0.476, *p* = 0.002) and after HD (session 2) (*R* ^2^ = 0.193, *p* = 0.034). Pulmonary congestion reflected by BLS was correlated to the global volume state reflected by BIA only in the second HD session (HD2) (*R* ^2^ = 0.374, *p* = 0.007). NT-proBNP levels and BLS were correlated before both sessions (*R* ^2^ = 0.421, *p* = 0.004, and *R* ^2^ = 0.505, *p* = 0.001, respectively). Systemic congestion was quite prevalent, as mean pre- and post-dialysis IVC dimensions and dynamics were quite elevated in both sessions, with a higher level of systemic congestion in the first HD session (diameter and collapsibility of 2.1 cm and 23%, and 2.01 cm and 19%, respectively) compared with the second session (1.98 cm and 17.5%, and 1.9 cm and 22%, respectively) without reaching statistical significance. IVC dimensions and global volume status measured by BIA were correlated in the second dialysis session (*R* ^2^ = 0.260, *p* = 0.031). No correlation was found between IVC dimensions and diastolic cardiac function (e/è ratio) parameters or with NT-proBNP levels. On day 30, BLS was significantly reduced in the active group, whereas no difference was found in the control group. However, no real impact was observed on IVC dimensions and dynamics or in total volume status by BIA.

**Conclusion:**

Pulmonary congestion is common in HD patients even after reaching their dry weight at the end of two consecutive sessions, and it is not correlated to systemic congestion, suggesting a complex multifactorial pathophysiology origin. Global volume status reflected by BIA and cardiac function are not always related to either systemic congestion represented by IVC dimensions or pulmonary congestion represented by BLS. Fluid redistribution anomalies may allow pulmonary congestion accumulation independently from systemic congestion and global volume status (non-cardiogenic pulmonary congestion). We recommend a personalised approach when managing HD patients by integrating systemic and pulmonary congestion parameters. Dry weight modification guided by repeat LUS may safely reduce pulmonary congestion. However, no impact was observed on systemic congestion or global volume status.

## Introduction

End-stage kidney disease (ESKD) patients treated with hemodialysis (HD) have a complicated and dynamic volume status. As their urine output is low or even absent, they accumulate fluids between their dialysis sessions. Usually, they follow a thrice-weekly HD planning, with variable inter-dialytic intervals (68 hours *vs.* 44 hours), which makes their volume status more complicated to evaluate.

This variable accumulation of fluid produces systemic and pulmonary congestion. Clinical examination is important to evaluate the signs of congestion; however, it is not accurate enough to provide an objective dry weight (the best-estimated weight where the patient has no congestion) to guide the hemodialysis treatment prescription (1).

One additional tool to evaluate systemic congestion is to measure inferior vena cava (IVC) diameters and dynamics. Global volume status may be assessed by bioelectrical impedance analysis (BIA). Lung ultrasound (LUS) is a reliable tool to detect and quantify pulmonary congestion (2).

Adding these tools to the standard of care to better establish the dry weight may be advantageous. However, currently, there is no simple clear protocol for integrating them into the clinical practice.

In addition, the correlation between the objective measures by these tools reflecting different aspects of congestion is not completely clear.

It was shown that pulmonary congestion assessed by a validated B-line score (BLS) using LUS is common among asymptomatic HD and peritoneal dialysis patients (3). Furthermore, the presence of pulmonary congestion in patients on maintenance HD, regardless of volume overload, is associated with adverse outcomes (3, 4).

The challenge is thus to establish an early diagnosis of PC at the bedside before symptoms appear to maintain a good quality of life and potentially reduce the risk of morbidity and mortality, in addition to preventing full development of pulmonary oedema. Fluid redistribution cannot be assessed by classically determined dry weight (DW). Thus, DW is less reliable in reducing pulmonary congestion, giving a place for a multifactorial management strategy guided by LUS (2).

LUS consists of detecting discrete laser-like vertical hyperechoic reverberation artefacts arising from the pleural line, extending to the bottom of the screen, namely, the B-lines. B-line counts represent a simple and reliable method to assess PC and evaluate effective water retention in the lung. A meta-analysis comparing LUS with chest X-ray suggests that B-line count is more sensitive than radiography in detecting pulmonary oedema and that it should be included as an additional diagnostic modality in patients presenting with acute dyspnoea (4).

Estimating the ideal weight of hemodialysis patients is still challenging for nephrologists, as the available tools to obtain such estimations are not accurate in reflecting the global volume state of the patient. Furthermore, there are limited approaches to evaluate congestion in different body compartments.

Understanding the relationship between different body compartment congestion using new tools may allow for better management of HD patients.

Our study aimed to examine the correlation between pulmonary congestion reflected by LUS, systemic congestion reflected by IVC, and global volume status reflected by BIA and investigate the impact of variable inter-dialytic intervals.

We examined the effect of simplified LUS-guided management on these parameters.

## Methods

We conducted a prospective randomized pilot study in 18 HD patients, which was preceded by an observational phase on the same patient group. All participants were recruited from our HD unit at Brugmann University Hospital.

The study received approval from the Research Ethics Committee of our hospital and was performed according to institutional procedures and the Declaration of Helsinki. All participants provided signed written informed consent before inclusion.

### Patient inclusion/exclusion criteria and clinical/biological data collection

Eighteen adult patients on maintenance HD for at least 3 months in our high-care unit were included. Patients diagnosed with interstitial lung disease or recent pneumonia, who had previous lung surgery, and or who had cancer were excluded.

Charts with the most current values available were reviewed to collect data, including demographics (age and sex), HD treatment parameters, cause of chronic kidney disease, laboratory parameters (serum urea, phosphate, albumin, and haemoglobin), body mass index (BMI), DW, weight before and after HD sessions, pre- and post-dialysis blood pressure, comorbidities such as diabetes and previous cardiovascular events, and antihypertensive therapy.

### Design of the observational phase of the protocol

**Figure 1:**
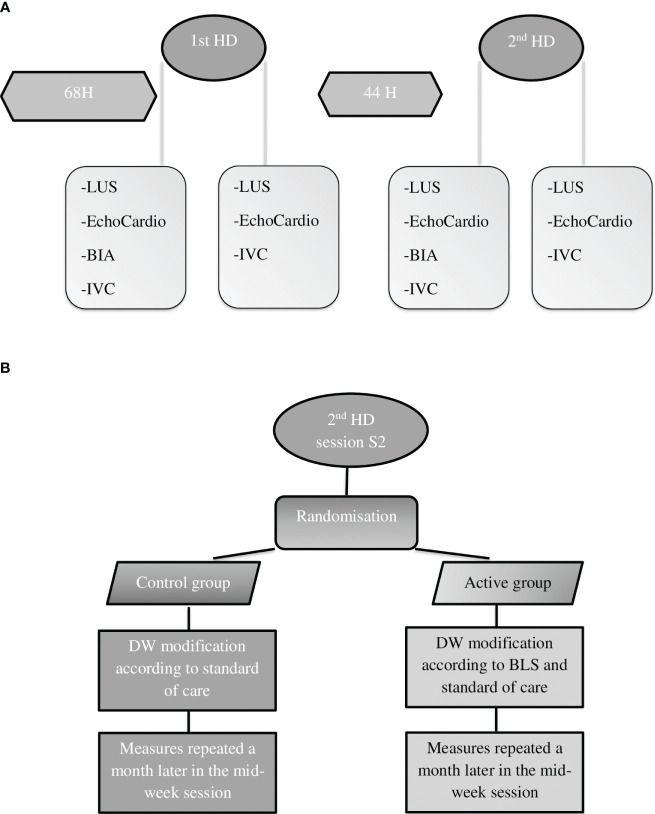
Study design of the observational phase **(A)** and the interventional phase **(B)**. LUS, lung ultrasound; BIA, bioelectrical impedance analysis; IVC, inferior vena cava; BLS, B-line score; DW, dry weight; h, hours.

As schematically illustrated in **Figure 1A**, all patients underwent LUS and echocardiography in a supine or near-supine position before and after their regularly scheduled first and second HD sessions of the week. All measurements were performed by the same operator at the bedside using the same ultrasound machine (T-Lite system, Sonoscanner, Meditor, La Wantzenau, France).

To quantify pulmonary congestion, an individual BLS was obtained according to the eight-site method by LUS. The cutoff for the B-line score was fixed at 0.54 line per zone (5). Lung ultrasound is useful for assessing the presence and severity of pulmonary congestion, but the most extensively validated 28-zone study is time-consuming. Among HD patients, four-, six-, and eight-zone lung ultrasound protocols were comparable with 28-zone studies for PC assessment (5).

Echocardiography was performed pre- and post-HD sessions 1 and 2 together with LUS using a T-Lite system, applying a standardized protocol including parasternal long- and short-axis views and apical four-chamber views. Cardiac systolic function was evaluated by measuring left ventricular ejection fraction (LVEF). Pulsed-wave Doppler assessment of mitral valve inflow was used to calculate the E/A ratio. Tissue Doppler velocities were measured at the medial and lateral mitral valve annular tissues to determine the e/è ratio, reflecting cardiac diastolic function. The diameter and dynamics of the IVC were also examined. Echocardiographic parameters were compared with the results of patients’ basic echocardiography, performed by a cardiologist within a year before the starting date of the study. LVEF and e/è were well correlated when basic echocardiography results were compared with the mean value of our six repeated measurements collected during the present study (*p* = 0.006, *R* ^2^ = 0.352, and *p* = 0.006, *R* ^2^ = 0.386, respectively) (As shown in **Table 2**).

In addition, BIA was performed before each HD session using a portable whole-body bioimpedance spectroscopy device (BCM, Fresenius Medical Care Deutschland GmbH, Biebesheim am Rhein, Germany). Serum N-terminal pro-brain natriuretic peptide type B (NT-proBNP) levels were obtained before each session.

### Design of the interventional phase of the protocol

After completion of the observational phase, patients were randomized into two groups:

1. Interventional arm group (“active group”): Dry weight was modified according to individual BLS obtained after the second HD session, considered as day 1, in addition to standard of care. Practically, each patient’s dry weight was reduced by 500 mg if the BLS was >0.54 line/zone. Another evaluation of the BLS was performed on day 15, where dry weight was also modified according to the same rule.
2. Control arm group (“control group”): Dry weight was modified according to the standard of care only.

The same measurements as those performed during the observational phase were repeated in the second HD session of the week on day 30 in both groups (**Figure 1B**).

Classical statistical methods (*t*-test and *Q^2^* test to test for differences, as appropriate) were applied using professional statistic software (Jamovi and SPSS).

## Results

The patient’s basic clinical and biological characteristics are summarised in **Table 1**.

**Table 1:** Patients’ basic and biological characteristics.

Variable	Value
Number	18
Age (year)	68 (24–88)
Female/male ratio	3/15
Diabetes, *n* (%)	6 (33%)
Hypertension, *n* (%)	16 (89%)
Heart failure, *n* (%)	3 (17%)
AVF, *n* (%)	10 (55%)
Central catheter, *n* (%)	8 (45%)
HD, *n* (%)	8 (45%)
HDF, *n* (%)	10 (55%)
Mean BMI, kg/m^2^ (min–max)	25.6 (15–34)
Haemoglobin mean, g/dl (min–max)	10.8 (7.4–12.7)
Kt/V, mean ( ± SD)	1.74 ( ± 0.38)
Dialysis vintage, mean (months)	65.9
Residual urine volume (ml)	437
Albumin (g/dl), mean ( ± SD)	40.6 ( ± 4.1)
Calcium (mmol/L), mean	2.36
Potassium (mmol/L), mean	5
Phosphorus (mmol/L), mean	1.53
Pre-dialysis urea (mg/dl), mean	123
Post-dialysis urea (mg/dl), mean	29.4

AVF, arteriovenous fistula; HD, hemodialysis; HDF, haemodiafiltration; BMI, body mass index.

### Observational phase

Pulmonary congestion was frequent both before and after dialysis in both sessions regardless of the inter-dialytic interval (pre-dialysis, 16±5.53, and post-dialysis, 15.3±6.63; pre-dialysis, 16.3±5.27, and post-dialysis, 13.6±5.83, respectively).

Systemic congestion was also frequent, as mean pre- and post-dialysis IVC dimensions and dynamics were quite elevated in both sessions, with a higher level of systemic congestion in the first HD session (diameter and collapsibility of 2.1 cm and 23%, and 2.01 cm and 19%, respectively) compared with the second session (1.98 cm and 17.5%, and 1.9 cm and 22%, respectively), without reaching statistical significance.

**Figure 2:**
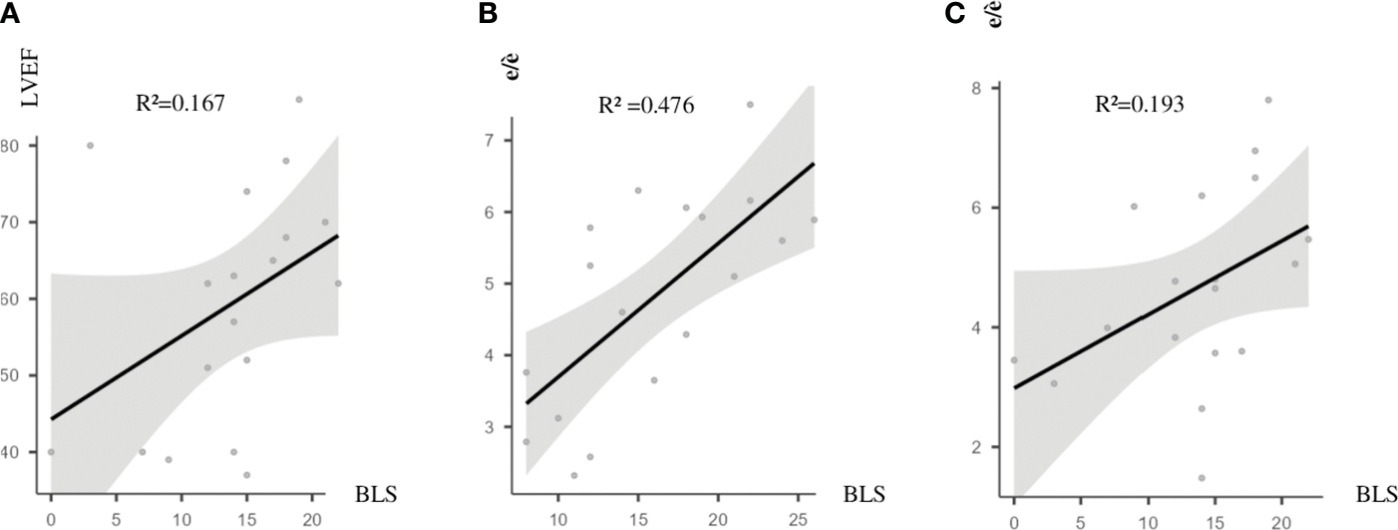
Correlations between PC (reflected by BLS) and cardiac functional markers. **(A)** BLS and LVEF after HD2; *R* ^2^ = 0.167, *p* = 0.046. **(B)** BLS and e/è before HD1; *R* ^2^ = 0.476, *p* = 0.002. **(C)** BLS and e/è after HD2; *R* ^2^ = 0.193, *p* = 0.034. BLS, B-line score; PC, pulmonary congestion; LVEF, left ventricular ejection fraction; HD2, second hemodialysis session; HD1, first hemodialysis session.

Systolic (left ventricular ejection fraction) and diastolic cardiac function (e/è ratio) parameters from one side and pulmonary congestion (BLS) from the other were not always correlated. BLS was correlated to the e/è ratio before HD (session 1) (*R* ^2^ = 0.476, *p* = 0.002) and after HD (session 2) (*R* ^2^ = 0.193, *p* = 0.034) (**Figure 2**).

**Figure 3:**
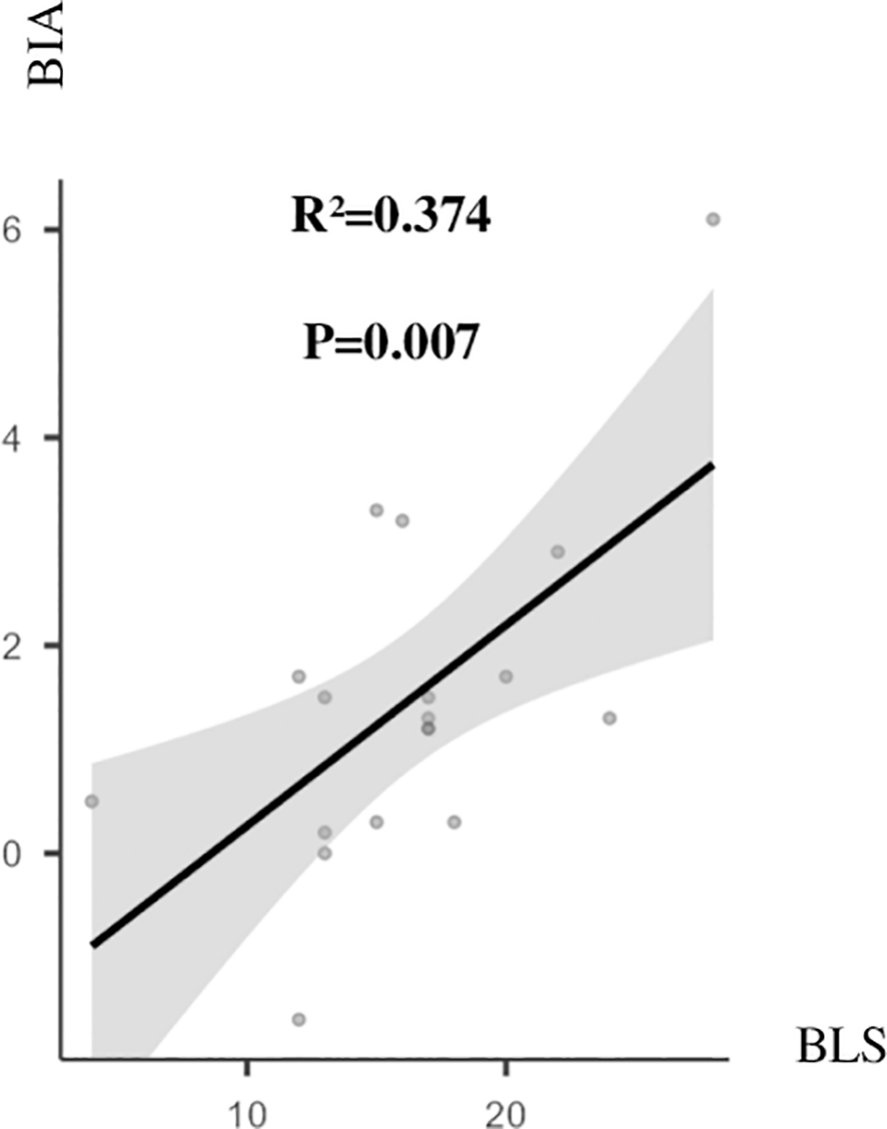
BLS and BIA before HD2. *R* ^2 =^ 0.374, *p* = 0.007. BIA, bioelectrical impedance analysis in litre; BLS, B-line score; HD2, second hemodialysis session.

Pulmonary congestion reflected by BLS was correlated to the global volume state reflected by BIA only in the second HD session (HD2) (*R* ^2^ = 0.374, *p* = 0.007) (**Figure 3**).

NT-ProBNP levels and BLS were correlated before both sessions (*R* ^2^ = 0.421, *p* = 0.004; *R* ^2^ = 0.505, *p* = 0.001, respectively).

IVC dimensions and global volume status measured by BIA were correlated in the second dialysis session (*R* ^2^ = 0.260, *p* = 0.031). No correlation was found between IVC dimensions and diastolic cardiac function (e/è ratio) parameters or with NT-proBNP levels.

No correlation was found between pulmonary congestion represented by LUS and systemic congestion represented by IVC.

Basic echocardiographic findings from cardiologists’ reports made in the year before the study were similar to our findings with no significant differences (**Table 2**).

**Table 2:** Basic and echocardiographic features.

	Basic EF	Mean EF	Basic e/è	Mean e/è
*N*	17	18	18	18
Mean	50.9	56.7	10.1	4.45
Median	51	56.6	9.13	4.32
Standard deviation	11.3	10.2	6.22	1.04

EF, ejection fraction.

### Interventional phase

**Figure 4:**
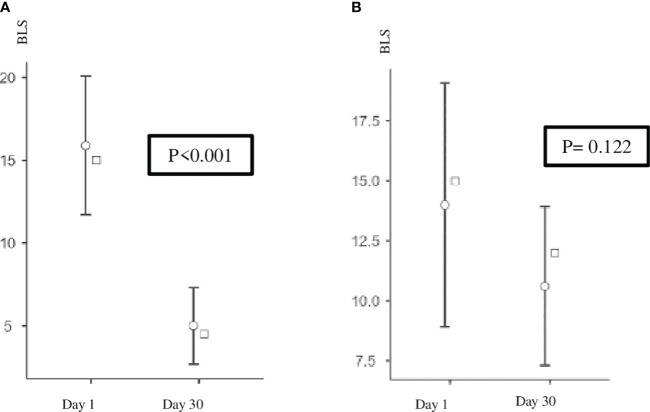
**(A)** Active group: BLS after HD on days 1 and 30. *p* < 0.001. **(B)** Control group: BLS after HD on days 1 and 30. BLS, B-line score; HD, hemodialysis.

On day 30, a significant reduction in BLS was observed before (17.4 *vs.* 8.5, *p* < 0.0001, effect size (ES) = 2.63) and after (13.3 *vs.* 5, *p* < 0.001, ES = 2.1) HD in the active group, whereas no difference was found in the control group before (14.9 *vs.* 12.1, *p* = 0.16) and after (14 *vs.* 10.6, *p* = 0.122) HD (**Figure 4**).

This reduction in pulmonary congestion in the active group was not associated with a statistically significant reduction in systemic congestion (IVC) or global volume status (BIA).

## Discussion

This study reveals that there is a weak correlation between systemic and pulmonary congestion in addition to volume status in hemodialysis patients.

Also, it shows that a LUS-guided management was able to reduce pulmonary congestion in a significant way. However, no real impact was observed on systemic congestion or global volume status.

Pulmonary congestion was quite frequent. It was reduced after the dialysis session by ultrafiltration (UF). However, it remained relatively high even when patients reached their estimated dry weight. This is in line with the work of Noble et al., who demonstrated that UF induces a concomitant reduction of the B lines during dialysis treatment (6).

Volume status measured by BIA was not always correlated to pulmonary congestion or systemic congestion. Volume redistribution through a damaged alveolar–capillary barrier may explain why pulmonary congestion estimated by LUS is not always related to body water volume estimated by BIA. This structure damage may be the result of inflammation, oxidative stress, or other causes related to uraemia. Interstitial space congestion caused by the chronic nature of ESKD may explain the weak correlation between the global volume status and systemic congestion.

Supporting our findings, two studies found that BLS and total body water by BIA measured together were very weakly associated (7, 8).

Along the same lines, studies conducted on patients with acute decompensated heart failure concluded that patients’ clinical improvement did not correlate with a change in their weight. This confirms the idea that symptoms resulting from volume expansion are secondary to redistribution rather than the accumulation of fluids (9).

Numerous trials and epidemiological studies have demonstrated the prevalence of pulmonary congestion in patients with chronic heart failure (HF). The *post hoc* analysis of the LUS‐HF trial revealed that up to 40% of patients considered “dry” according to pulmonary auscultation presented LUS‐evidenced pulmonary congestion at hospital discharge. These patients also experienced worse prognoses at 6‐month follow‐up (10).

In the interventional phase, our simplified LUS-guided management was able to reduce pulmonary congestion in a significant way. This reduction in PC was not associated with a reduction in total body volume estimated by BIA or systemic congestion represented by IVC, which encourages us to investigate further the intercommunication between the interstitial volume expansion, vascular volume expansion, and pulmonary alveolar water and how these volumes interact. We hypothesize that chronic interstitial volume expansion caused by ESKD is difficult to reverse and may even be irreversible, whereas vascular volume expansion and even more pulmonary alveolar water are easier to manage and reduce. This pathophysiological hypothesis may be one of the factors explaining why a slight reduction in the dry weight guided by BLS compared with the standard of care has a real impact on pulmonary congestion.

This congestion in multiple compartments (systemic, interstitial, and pulmonary) and fluid movement speed between them may be different from one patient to another, which makes the use of every available tool to evaluate every space and its dynamic a crucial element to reach a personalized approach in the management of HD patients on a case-by-case basis.

Building a protocol that integrates these tools will possibly provide better objective markers to establish the best management for HD patients.

In conclusion, the correlation between pulmonary congestion, systemic congestion, and global volume status in hemodialysis patients is weak and independent of variable inter-dialytic intervals. Our simplified LUS-guided management approach was very useful in reducing pulmonary congestion when it was added to the standard of care. However, the effect on systemic and global volume status was weak, encouraging us to find a more complete protocol integrating BIA and IVC in the management of hemodialysis patients.

This study has limitations. As a pilot study, it had a low sample size and a monocentric nature.

## Data availability statement

The datasets presented in this study can be found in online repositories. The names of the repository/repositories and accession number(s) can be found below: DOI 10.6084/m9.figshare.24099672.

## Ethics statement

The studies involving humans were approved by the Brugmann University Hospital ethics committee. The studies were conducted in accordance with the local legislation and institutional requirements. The participants provided their written informed consent to participate in this study.

## Author contributions

SK: Conceptualization, Data curation, Formal analysis, Investigation, Methodology, Writing – original draft, Writing – review & editing. BP: Data curation, Software, Writing – review & editing. MM: Writing – review & editing. FC: Writing – review & editing. JN: Conceptualization, Methodology, Supervision, Writing – review & editing.
